# Elderly Subjects Supplemented with L-Glutamine Shows an Improvement of Mucosal Immunity in the Upper Airways in Response to Influenza Virus Vaccination

**DOI:** 10.3390/vaccines9020107

**Published:** 2021-01-31

**Authors:** Vitória Paixão, Ewin B. Almeida, Jonatas B. Amaral, Tamaris Roseira, Fernanda R. Monteiro, Roberta Foster, Adriane Sperandio, Marcelo Rossi, Gislene R. Amirato, Carlos A. F. Santos, Renier S. Pires, Fabyano B. Leal, Edison L. Durigon, Danielle B. L. Oliveira, Rodolfo P. Vieira, Mauro Vaisberg, Juliana M. B. Santos, André L. L. Bachi

**Affiliations:** 1 Department of Otorhinolaryngology, ENT Lab, Federal University of São Paulo (UNIFESP), São Paulo 04021-001, Brazil; v.paixao@unifesp.br (V.P.); ewin.almeida@unifesp.br (E.B.A.); jbamaral@unifesp.br (J.B.A.); tamaris.pavao@soufamesp.com.br (T.R.); fernanda.monteiro@soufamesp.com.br (F.R.M.); roberta.foster@timefamesp.com.br (R.F.); mrossi@dim.fm.usp.br (M.R.); prof.gislene@hotmail.com (G.R.A.); vaisberg.mauro@gmail.com (M.V.); abachi@unifesp.br (A.L.L.B.); 2 Method Faculty of São Paulo (FAMESP), São Paulo 04046-200, Brazil; ane.sperandio@gmail.com; 3 Department of Medicine, Geriatry, Paulista School of Medicine (EPM), São Paulo 04023-062, Brazil; freitas.carlos@uol.com.br; 4 Post-Graduation Program in Health Science, Santo Amaro University (UNISA), São Paulo 04743-030, Brazil; rspiress@yahoo.com.br; 5 Institute of Biomedical Science, University of São Paulo (USP), São Paulo 05508-060, Brazil; fabyo_leal@icb.usp.br (F.B.L.); eldurigo@usp.br (E.L.D.); danibruna@gmail.com (D.B.L.O.); 6 Scientific Platform Pasteur, University of São Paulo (USP), São Paulo 05508-060, Brazil; 7 Hospital Israelita Albert Einstein, São Paulo 05652-900, Brazil; 8 Brazilian Institute of Teaching and Research in Pulmonary and Exercise Immunology (IBEPIPE), São Paulo 12245-520, Brazil; rpvieira@unifesp.br; 9 Post-Graduation Program in Bioengineering and Biomedical Engineering, Universidade Brasil, São Paulo 15600-000, Brazil; 10 Post-Graduation Program in Science of Human and Rehabilitation, Federal University of São Paulo (UNIFESP), Santos 11015-020, Brazil

**Keywords:** L-glutamine, influenza virus, vaccine, antibodies, immunoglobulin, cytokines

## Abstract

Background: Although glutamine is able to improve the immune response, its action in the upper airway immunity against the influenza virus vaccine remains unclear. Therefore, we aimed to evaluate the L-glutamine supplementation effect on the mucosal immune/inflammatory response of elderly subjects vaccinated against the influenza virus. Methods: Saliva sampling from 83 physically active elderly volunteers were collected pre- and 30 days after influenza virus vaccination and supplementation with L-glutamine (Gln, *n* = 42) or placebo (PL, *n* = 41). Results: Gln group showed higher salivary levels of interleukin (IL)-17, total secretory immunoglobulin A (SIgA), and specific-SIgA post-vaccination than values found pre-vaccination and in the PL group post-vaccination. Whereas higher salivary levels of IL-6 and IL-10 were observed post-vaccination in the Gln group, IL-37 levels were lower post-vaccination in both groups than the values pre-vaccination. Tumor necrosis factor (TNF)-α levels were unchanged. Positive correlations between IL-6 and IL-10 were found in all volunteer groups pre- and post-vaccination and also between IL-17 and IL-6 or IL-10 in the Gln group post-vaccination. A negative correlation between IL-37 and IL-10 was found pre- and post-vaccination in the PL group. Conclusion: Gln supplementation was able to modulate salivary cytokine profile and increase SIgA levels, both total and specific to the influenza virus vaccine, in physically active elderly subjects.

## 1. Introduction

The influenza virus infection is a challenge for the public health system due to its high rates of hospital admissions and deaths, especially in children under two years old and the elderly population [1,2]. Several factors influence the influenza virus infection outcome, which includes the interaction between the virus and the host cell response, secondary bacterial infections, and some comorbidities, such as cerebrovascular disease, ischemic heart disease, and diabetes, besides the presence of immunodeficiencies, particularly immunosenescence [1,3].

It is broadly known that aging corresponds with decreased immune responses, a situation named immunosenescence, which leads the elderly subjects to present a lower capacity to protect themselves against infections and also respond to immunization. Regarding the vaccine-induced immunogenicity, which is defined as “the strength or magnitude of an immune response” [4], it was reported that the elderly population shows a magnitude of the influenza virus vaccination response of only 30–40% [5,6].

Studies have demonstrated that a remarkable factor with negative impacts in response to vaccination in older adults is the phenomenon of inflammaging, which is characterized by a low-grade chronic systemic inflammation associated with aging. With inflammaging, some classical proinflammatory cytokines, such as the tumor necrosis factor-α (TNF-α) as well as interleukin-1 (IL-1) and IL-6, are increased and can influence accelerating the immunosenescence [7,8,9,10].

Previously, our group reported that physically active elderly subjects showed improvements in the specific-antibody immune response to influenza virus vaccination, as well as lower systemic proinflammatory cytokines levels as compared to sedentary elderly subjects [11]. In order to amplify our understanding of interventions that can mitigate the effect of aging on the immune response against the influenza vaccine, beyond the regular practice of exercise training, an adequate nutritional balance with supplementation including proteins as well as amino-acids has been helpful to achieve this goal [12,13]. In this respect, recently, our group was able to demonstrate that a group of elderly subjects supplemented with L-glutamine showed enhancement of both humoral and cellular immunity against influenza virus vaccination [14]. Furthermore, we also showed that oral L-glutamine supplementation presents a notable capacity to modulate oxidative stress and inflammation and to improve salivary redox indexes in the same group of elderly subjects [15]. It is paramount to highlight that glutamine (Gln) is a non-essential amino-acid preferentially produced by the musculoskeletal tissue that presents pleiotropic actions in many systems, especially in the immune system. Based on the literature, the Gln is considered an essential nutrient for the immune cells, mainly by its use as a source of metabolic intermediates, besides a substrate for nucleotide synthesis. Interestingly, it has been reported that the Gln consumption by the immune cells can be greater than glucose in order to guarantee adequate immunity against pathogens through its capacity of modulating macrophages, lymphocytes, and neutrophils functions [16,17,18,19,20]. However, despite the fact that the Gln has a singular property in the immune system and also that there are many studies that address the effects of the elderly immune response to the influenza vaccine [11,17,21], there is a lack of studies that aimed to evaluate the action of Gln in this context [14].

Although systemic evaluations are widely used to study the immune responses and can contribute to enhancing the knowledge about immunity, nowadays, the opportunity to evaluate mucosal immunity, particularly through saliva, has rendered attention and can be useful not only to improve the understanding of how mucosal immunity can be stimulated and respond to the vaccination, especially in terms of respiratory viruses but also to monitor both human health and illness status [22,23].

Based on these pieces of information, the aim of this study was to investigate the effect of oral L-glutamine supplementation in the upper airways mucosal immunity of a group of physically active elderly subjects vaccinated against the influenza virus through immune/inflammatory salivary biomarkers, such as total and specific-secretory immunoglobulin A (SIgA) for the vaccine, as well as pro- and anti-inflammatory cytokines.

## 2. Materials and Methods

### 2.1. Subjects and Study Design

**Figure 1:**
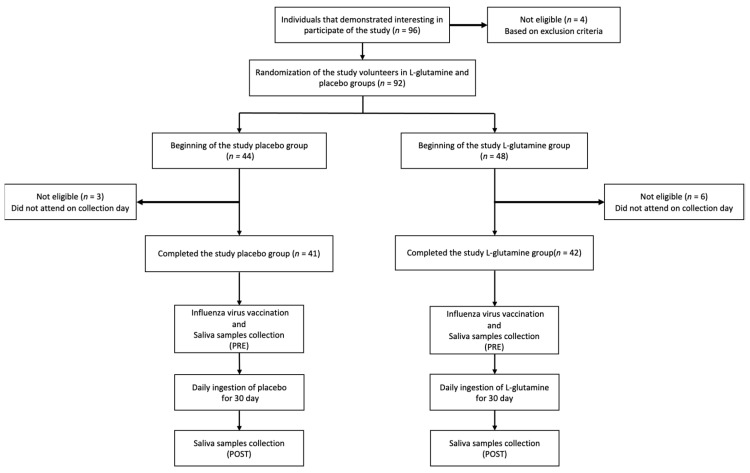
Flow diagram and experimental design of the study.

Eighty-three elderly subjects, with ages between 60–85 years, both women (*n* = 65) and men (*n* = 18), participated voluntarily in the study, as shown in the flow diagram in Figure 1. All volunteers were selected and recruited from the Discipline of Geriatrics and Gerontology belonged to the Federal University of São Paulo (UNIFESP). It is worth mentioning that the overwhelming majority of them have participated in other studies recently published by our group [14,15]. None of them took any antioxidant/multivitamin supplements and had a diet with a protein intake of more than 1.75 g/kg body mass at the time of the study; moreover, no participants used anti-inflammatory drugs in the last two months. In terms of clinical and physical examinations, none of them had HIV and/or another chronic infection, neoplasia, renal, liver, and/or neurological diseases, type I diabetes, nor any other diseases that restrained the practice of physical activity (exclusion criteria). All volunteers recruited gave written informed consent by signing the informed consent form previously approved by the National Research Ethics Committee (number CAEE:218170619.3.0.000.5505) and by the Ethics Committee of the Federal University of São Paulo (approval number 3.623.247) after they received the information regarding the risks and benefits of the study. It is important to mention that the study development was in agreement with the Declaration of Helsinki. According to what is demonstrated in Figure 1, saliva samples were obtained on two different time points: before (pre) and 30 days after (post) the supplementation and influenza virus vaccination.

### 2.2. Anthropometric Characteristics and Nutritional and Physical Activity Evaluations

Data regarding height, weight, body mass index (BMI), and body composition by bioimpedance (BIOSCAN 920-2-S Maltron International Limited, UK) were assessed. In addition, data concerning the nutritional consumption of proteins and antioxidants per day were obtained by the Food Frequency Questionnaire (FFQ) and the physical activity level was obtained by the International Physical Activity Questionnaire (IPAQ), validated prior for the Brazilian population [24,25].

### 2.3. L-Glutamine Supplementation

The volunteers participating in this study were separated into two groups: the L-glutamine group (Gln, *n* = 42) and the placebo group (PL, *n* = 41), following the same supplementation schedule previously reported by our group [15]. Briefly, the Gln group was supplemented with 0.3 g/kg/day of L-glutamine (Tongliao Meihua Biological Sci-Tech Co. Ltd., Tongliao, China) plus 10 g/day of maltodextrin (PR Netto Indústria e Comércio de Alimentos Ltd.a., São Paulo, Brazil), while the PL group were supplemented with 10 g/day of maltodextrin only. The supplementations were provided in sachets and all volunteers were instructed to ingest one dose immediately after its dilution in 250 mL of water for 30 consecutive days. It is noteworthy to clarify that, in agreement with the literature, a Gln adjusted dose of <1.0 g (usually 0.3–0.5 g) per kg of bodyweight is safe and tolerable for both healthy and ill populations [16,26,27,28,29]. In addition, if any gastrointestinal disturbance occurred, the volunteers were instructed to interrupt the supplementation immediately.

### 2.4. Influenza Virus Vaccination

All volunteer groups received the fragmented and inactivate trivalent seasonal influenza virus vaccine available in 2019 by the Butantan Institute (Sao Paulo, Brazil), which was the official Brazilian manufacturer for the influenza virus vaccine. This vaccine was composed of two types of influenza A virus [A/Michigan/45/2015 (H1N1) and A/Switzerland/8060/2017 (H3N2)] and a type of influenza B virus (B/Colorado/06/2017).

### 2.5. Detection of the Presence of Influenza Virus in the Upper Respiratory Tract by qRT-PCR

In order to verify whether the volunteers presented influenza virus in the upper respiratory tract on the day of sample collection (both pre- and post-vaccination and supplementation), a singleplex real-time reverse transcription-polymerase chain reaction (qRT-PCR) assay panel was used. By saliva samples and the qRT-PCR assay, we were able to detect and identify seasonal influenza A virus (sIAV), pandemic influenza A H1N1 virus (pH1N1), and influenza B virus (IBV), according to what was described by the Center Disease Control—GA/USA (https://www.cdc.gov/flu/professionals/diagnosis/molecular-assays.htm). The gene of influenza virus types A and B were obtained through the total nucleic acid (RNA and DNA) extraction by using the MVP II Kit, following the manufacturer’s’ instructions, in a semi-automated MegMax extraction machine (Applied Biosystems Inc., EUA). After that, the detection of viral RNA was carried out using the followed sequence of primers (influenza A- Forward- 5′ GACCRATCCTGTCACCTCTGA C 3′; Reverse- 5′AGGGCATTYTGGACAAAKCGTCTA3′; influenza B- Forward- 5′GGAGCAACCAATGCCAC 3′; Reverse- 5′GTKTAGGCGGTCTTGACCAG 3′) and probes (influenza A- Probe1- 5′ FAM-TGC AGT CCT CGC TCA CTG GGC ACG-BHQ1-3′; influenza B- Probe1 5’-(FAM)-ATAAACTTTGAAGCAGGAAT-(MGB)-3’), and also by using the AgPath-ID One-Step RT-PCR Kit (Applied Biosystems Inc., EUA) on an ABI 7500 SDS real-time PCR machine (Applied Biosystems, Weiterstadt, Germany). As a positive control, we used the vaccine strain A/Michigan/45/2015 (H1N1) and A/Switzerland/8060/2017 (H3N2 and B/Colorado/06/2017, from Butantan Institute.

### 2.6. Collection of Saliva Samples

Samples of saliva were collected at two different time points: pre (before) and 30 days after (post) supplementation and vaccination. As previously reported by Almeida et al. [15], 2 mL of saliva was collected directly in sterile 15 mL Falcon^®^ tubes and kept refrigerated until their centrifugation at 3000 rpm for 5 min. After that, 400 μL of supernatant was transferred to 1.5 mL microtubes and kept frozen at −80 °C. Samples of saliva containing blood were discarded and a new saliva collection was carried out. No buffer or preservative was used.

### 2.7. Determination of Total and Specific Secretory Immunoglobulin A (SIgA) for the Influenza Virus Vaccine

The salivary concentration of total secretory immunoglobulin A (SIgA) was determined by a commercial ELISA test (Elabscience Biotechnology Co. Ltd., Houston, TX, USA), following the manufacturer’s instructions. In relation to the analysis of the specific-SIgA response for the influenza virus vaccine, an ELISA “in house” test was carried out in agreement with Bachi et al. [11]. Briefly, the influenza virus vaccine was initially diluted (0.18 μg/mL) in 0.1 M carbonate-bicarbonate buffer (pH 9.6) and used to coat the high-binding microtiter plates, overnight at 4 °C. After the blocking step, the saliva samples were diluted 1:10,000 in PBS containing Tween (0.1%) and 0.25% BSA (PBS-T-BSA). The secondary antibody, a peroxidase-conjugated anti-human IgA (Sigma, St. Louis, MO, USA), was diluted 1:4000 in PBS-T-BSA. Absorbance was read at 450 nm on a microplate reader (Multiskan Sky Microplate Spectrophotometer ThermoFisher, Waltham, MA, USA).

### 2.8. Determination of Salivary Cytokines

Salivary cytokine concentrations were determined by using commercial ELISA kits. The following were assessed: tumor necrosis factor-α (TNF-α), interleukins (IL) 17 (IL-17), IL-37 (R&D System, Minneapolis, MN, USA), IL-6, and IL-10 (Invitrogen by Thermo Fisher Scientific, Vienna, Austria) in accordance with the manufacturer’s instructions. Cytokine concentration was calculated from standard curves that should present the correlation coefficients in the range of 0.95 to 0.99, besides intra- and inter-assay coefficients of variance in 3–5% and 8–10%, respectively. It is noteworthy to clarify that the cytokine concentration was normalized using the total protein concentration, which was determined by the Bradford method [30].

### 2.9. Statistical Analysis

Variables data obtained in the present study were initially evaluated against deviations of normality hypothesis by the Shapiro-Wilk test and also the homogeneity of variance was evaluated by the Levene test.

Parametric variables are shown as mean and standard error and non-parametric variables are shown as the median and interquartile interval. In this respect, the results of total SIgA and specific-SIgA for influenza virus vaccine levels, as well as TNF-α, showed deviations from normality; thus, these variables were analyzed by Mann–Whitney and Friedmann tests with a Müller-Dunn *post hoc* test to evaluate whether the differences of variance between the supplementation time points in the elderly groups were significant. The other variables were evaluated by t-Student and two-way ANOVA test for repeated measures with Student-Newman-Keus *post hoc* test.

In addition, Pearson’s product-moment correlation coefficient analysis was used to identify the occurrence of significant correlations between the salivary cytokine levels.

The significance level was *p* < 0.05 and the statistical analysis were performed with GraphPad Prism 8.1.2 software.

## 3. Results

Table 1 shows the data concerning the anthropometric characteristics, the number of elderly women and men participating in the study, and the physical activity level of volunteers from the PL and Gln groups. No differences were found in all these parameters between the volunteer groups.

In addition, it is also important to point out that none of the volunteers showed the presence of the influenza virus in upper airways mucosa, evaluated by qRT-PCR, both before and after 30 days of vaccination and supplementation period.

### 3.1. Gln Supplementation Enhances the Salivary SIgA Levels after the Vaccination

**Figure 2:**
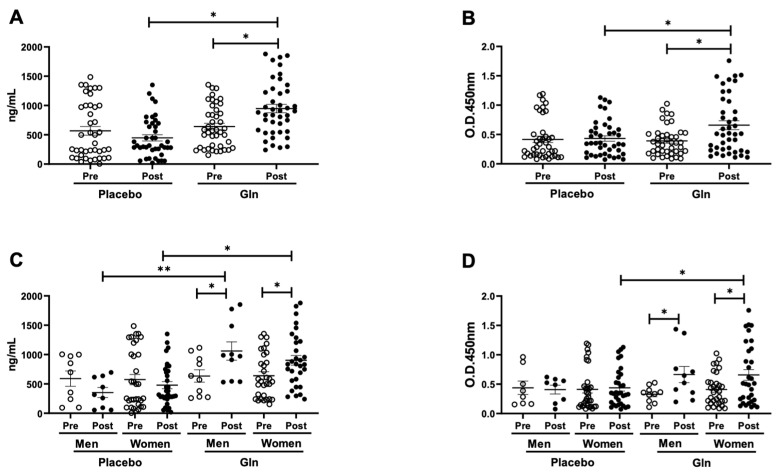
Salivary concentration of total SIgA (**A**,**C**) and specific-SIgA for the influenza virus vaccine (**B**,**D**) in the elderly subjects groups supplemented with placebo (PL) or L-glutamine (Gln) before (pre) and 30 days after (post) the vaccination and supplementation. Figure (**A**,**B**) show the data obtained from the volunteers not separated by sex, whereas (**C**,**D**) show the data obtained from the separation of the elderly volunteers by sex (women and men). Data were analyzed statistically using the Friedman test with a Müller-Dunn post-test and were presented as mean and standard deviation (SD) with a risk value of 5% (*p* < 0.05). ∗ *p* < 0.05, ∗∗ *p* < 0.01.

In Figure 2 is showed the salivary levels of total secretory IgA (SIgA, Figure 2A,C) and specific-SIgA for the influenza virus vaccine (Figure 2B,D). It is possible to observe that both the levels of total SIgA (Figure 2A) and specific-SIgA (Figure 2B) in saliva were higher in the Gln group post-vaccination than the values found before the vaccination (pre, *p* < 0.05) and also higher than the values observed in the PL group post-vaccination (*p* < 0.05). In addition, when the volunteer groups were separated by sex (women and men), higher total SIgA levels in saliva (Figure 2C) were observed in the elderly women and men post-supplementation with Gln than the values found before (pre) and also in the elderly women and men supplemented with placebo. In relation to the specific-SIgA for the influenza virus vaccine (Figure 2D), higher levels were found in elderly women and men post-supplementation with Gln than the values before (pre), but only the elderly women group supplemented with Gln presented significantly increased levels as compared to the elderly women group supplemented with placebo.

### 3.2. Gln Supplementation Modulates the Salivary Cytokines Levels

As previously mentioned, the results obtained in the evaluation of cytokines levels in the saliva samples were normalized by the total protein concentration (µg/mL), which showed a similar concentration (*p* > 0.05) not only between the groups but also in the time points analyzed (Placebo group—Pre = 20.21 ± 11.08 and Post = 20.24 ± 10.90; Gln group—Pre = 21.87 ± 10.97 and Post = 20.38 ± 10.87).

**Figure 3:**
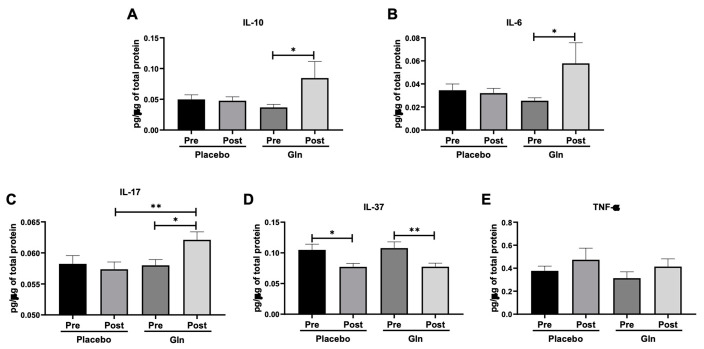
Salivary concentration of IL-10 (**A**), IL-6 (**B**), IL-17 (**C**), IL-37 (**D**), and TNF-α (**E**) in the elderly subject groups supplemented with placebo or L-glutamine (Gln) before (pre) and 30 days after (post) the vaccination and supplementation. Data were analyzed statistically using the Friedman test with Müller-Dunn post-test and were presented as mean and standard deviation (SD) with a risk value of 5% (*p* < 0.05). ∗ *p* < 0.05; ∗∗ *p* < 0.01.

Figure 3 shows that salivary levels of IL-10 (Figure 3A) and IL-6 (Figure 3B) were higher post-vaccination in the Gln group than the values before (pre, *p* < 0.05), whereas the levels of IL-17 (Figure 3C) in this group were higher post-vaccination at the same time point not only than before (pre, *p* < 0.05) but also as compared to the values found in the PL group post-vaccination (*p* < 0.01). In a different way, the levels of IL-37 (Figure 3D) were lower post-vaccination in both volunteer groups than the values before (pre, PL = *p* < 0.05 and Gln = *p* < 0.01). No differences were observed in the levels of TNF-α (Figure 3E).

### 3.3. Correlation Analysis between the Salivary Cytokine Levels Showed Significant Differences

**Figure 4:**
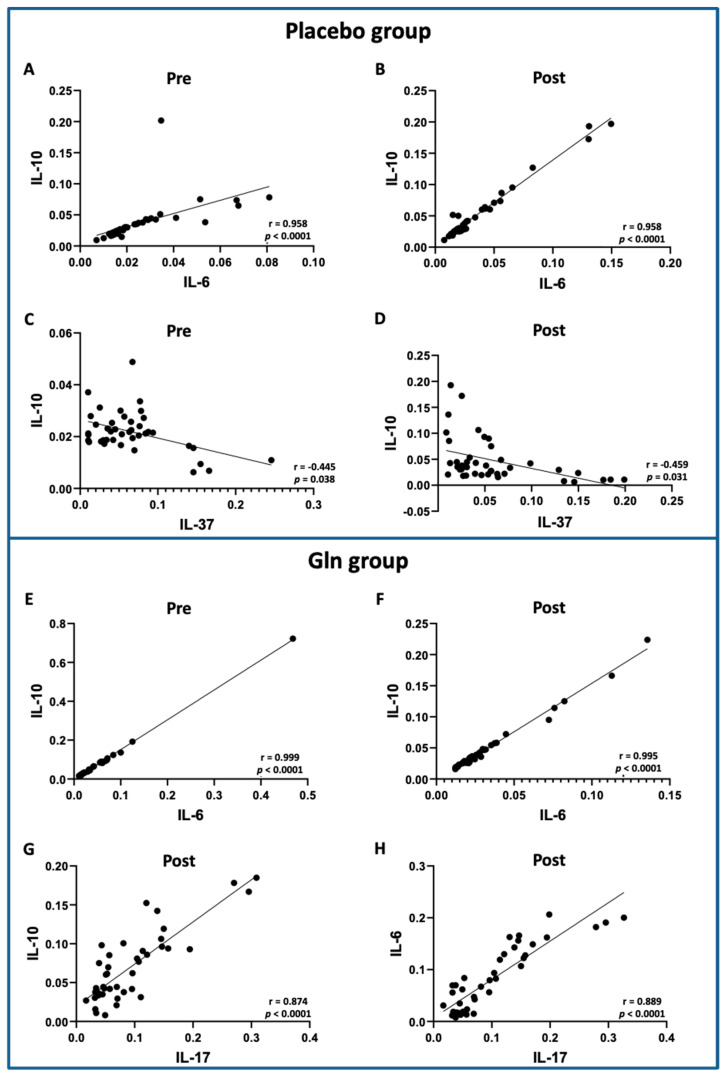
Significant results obtained by Pearson’s correlation coefficient analysis. Upper panel presents the positive significant correlations between IL-6 and IL-10 pre- (**A**) and post-supplementation and vaccination (**B**), as well as the negative significant correlation between IL-10 and IL-37 pre- (**C**) and post-supplementation and vaccination (**D**) in the placebo group. The lower panel presents the positive significant correlations between IL-6 and IL-10 pre- (**E**) and post-supplementation and vaccination (**F**), as well as the positive significant correlations between IL-17 and IL-10 (**G**) or IL-6 (**H**) post-supplementation and vaccination in the Gln group.

Figure 4 shows the significant results obtained in the correlation analysis of the salivary cytokine levels found in elderly subjects supplemented with placebo or L-glutamine (Gln) before (pre) 30 days after (post) influenza virus vaccination and supplementation. Positive correlations between IL-6 and IL-10 were found in both volunteer groups, pre (placebo A, Gln E) and post (placebo B, Gln F), besides other positive correlations between IL-17 and IL-10 (Figure 4G) or IL-6 (Figure 4H) post-vaccination and supplementation in the Gln group. In addition, negative correlations were found between IL-37 and IL-10 pre- (Figure 4C) and post-vaccination and supplementation (Figure 4D) in the placebo group only. In addition, all the results obtained in this analysis were presented in the supplementary material, in Table S1. 

## 4. Discussion

**Figure 5:**
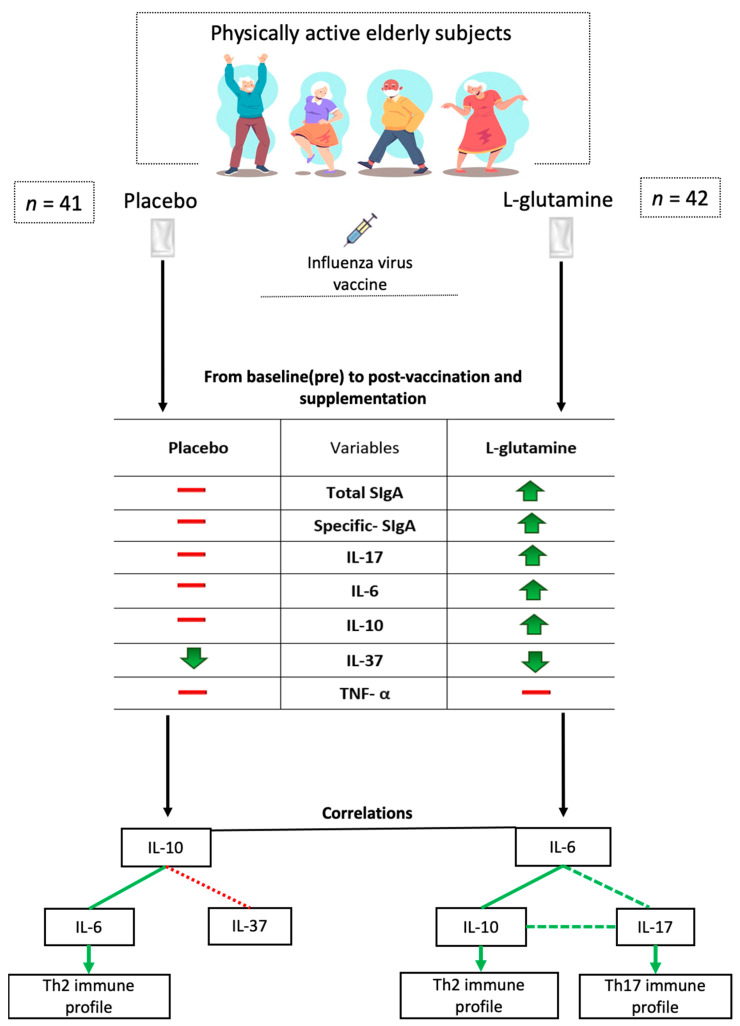
Representative illustration of the main findings of the study. Saliva sampling from all physically active elderly subjects was obtained before and 30 days after influenza virus vaccination and placebo or L-glutamine (Gln) supplementation. In the box, the green arrows indicate that the immunological parameter evaluated in the saliva increased post-vaccination and supplementation in comparison to baseline (pre) values, whereas the red lines indicate that the values remained unchanged during the study. In the flow chart, the constant green line indicates that a positive significant correlation was found both before and after influenza virus vaccination and supplementation; the dashed green line indicates that a positive significant correlation was found post-vaccination and supplementation, whereas the red dotted line indicates that a negative significant correlation was found pre-vaccination and supplementation. Abbreviation: IL = interleukin; SIgA = secretory immunoglobulin A; Th = T helper.

Our results showed, for the first time, that Gln supplementation was able to increase the levels of not only total SIgA but also specific-SIgA for the antigens of the influenza virus vaccine as well as modulating the cytokine profile in physically active elderly subjects, as summarized in Figure 5.

As cited before, the immunosenescence is a phenomenon closely associated with a lower influenza virus vaccine immunogenicity, which, in a general way, is around 30–40% [31]. Studies demonstrated that the elderly subjects vaccinated show reduced levels of IgG and IgA, presenting the peak of antibodies later and maintaining these titers for a short time as compared to the young subjects [32,33]. Particularly, the vaccine-immunogenicity is mainly evaluated by serum specific antibody (both IgM and IgG) responses. However, it has been suggested that immune responses derived-immunizations can be evaluated by the presence of specific-SIgA in saliva. Corroborating this suggestion, Ivanov et al. [34] pointed out that the use of specific salivary SIgA can be useful for the assessment of mucosal immunity in populations immunized with Sabin vaccine (poliovirus).

At this point, it should be emphasized that currently, the use of saliva has been suggested in different contexts, such as research, diagnosis, and clinical, and patient’s follow-up has been highlighted, mainly by some potential benefits that include non-invasive and easy collection, safety for the handler, and that it does not clot like blood [35,36,37,38]. In terms of the mucosal immunity of the upper airways, the signature of saliva as a representative biological fluid of this context can be proved through SIgA evaluations [34].

In accordance with the literature, the SIgA represents the “first line of defense” in the mucosa due to its capacity to directly inhibit many pathogenic agents [39,40]. It is noteworthy to mention that a reduction in the airways mucosal SIgA levels is associated with a higher risk of upper respiratory tract infection (URTI) development [39] mainly by a respiratory virus [41], such as rhinovirus, respiratory syncytial virus, and influenza virus [42]. In addition, it was reported that lower SIgA levels are associated with illness severity [43].

Recently, Russel and colleagues [23] proposed that the comprehension of mucosal immunity role and, particularly, SIgA, in the context of COVID-19 can illuminate the way in order to elucidate the differences in the outcome of SARS-CoV-2 infection. In this sense, the same authors declare that the mucosal immunity assessment, including T cell response and SIgA evaluations, can be helpful for diagnostic, therapeutic, or prophylactic purposes.

It is paramount to highlight that SIgA is rapidly produced and secreted in the mucosa (1 day) after an antigenic challenge, whereas serum IgA can often be detected after 3–5 days. Therefore, it is clear that SIgA can putatively provide an early and better picture of airway infections than serum IgA [30,44]. Furthermore, concerning the literature, the amount of SIgA in airways is between 70 to 850 micrograms per milliliter (ug/mL) [45]. In order to clarify the importance of SIgA released by the nasopharyngeal lymphoid tissue in terms of respiratory virus, it was demonstrated that children that have undergone tonsillectomy presented a significant reduction in the levels of specific IgAs for the poliovirus in airway secretions than children with tonsils [46].

Interestingly, regarding the impact of aging in the total salivary SIgA levels, it has been shown that its levels may increase with age, which could guarantee an efficient defense of airways against infections [47,48,49]. However, this increase in total SIgA observed during aging is not able to protect the elderly subjects, since they are the most susceptible population to present respiratory infections [11]. Another point that claims attention is related to the fact that there are differences in the immune responses between elderly women and men. Based on the data presented by Flanagan and colleagues [50], differences in the genetic aspects, sex hormones, diet, occurrence of chronic infection, and inflammation, as well as acceptance and access of vaccines across the life course, can contribute to the different immune responses between aged women and men.

Particularly, in relation to the antibody response to vaccines between elderly women and men, it was observed that elderly men presented higher antibody levels to the tetanus-diphtheria, pertussis, and pneumococcal vaccines than women, whereas elderly women presented higher antibody levels to the trivalent inactivated influenza virus vaccine than men [51]. In a similar way to the above described, studies have demonstrated significant differences in the total SIgA levels in saliva between aged women and men; however, there is currently no consensus [52].

Thus, beyond the assessment of total salivary SIgA, it is very important to evaluate the levels of specific-SIgA against the main pathogenic agents involved in respiratory infections. In this sense, the novelty of this study was to demonstrate that the Gln supplementation was able to increase the salivary levels of total SIgA and, most importantly, the specific-SIgA against the influenza virus vaccine. Furthermore, although it has been observed that elderly women present a better antibody response in terms of the influenza virus vaccine, in this study, we did not find significant differences in the levels of total SIgA nor specific-SIgA for the influenza virus vaccine in the saliva of elderly women and men. In this respect, we can putatively suggest that the fact that all volunteer participants in this study had a physically active lifestyle could mitigate the differences of gender in the vaccine-induced immunogenicity [53], leading to an improvement of the immune response against influenza vaccination both in elderly women and men. Moreover, it is of utmost importance to mention that the salivary levels of specific-SIgA found here were not impacted by the presence of the influenza virus, since the qRT-PCR analysis did not show the infection by this virus in the sampling collection. Thus, we can putatively affirm that the specific-SIgA response was derived from the immune response against the influenza virus vaccine.

In spite of the literature showing a gap of data concerning the effects of Gln supplementation in the SIgA production, particularly in the mucosa of the upper airways, recently, our group was able to demonstrate that elderly practitioners of combined exercise training that were supplemented with Gln presented an improvement of some antioxidant parameters [15]. Corroborating this beneficial effect of Gln in elderly subjects, another study developed by our group, also recently published [14], in which the same group of elderly practitioners of combined exercise training was evaluated, the Gln supplementation was able to increase the levels of specific antibodies, particularly IgM and IgA, for the influenza virus vaccine. Although we cited that the overwhelming majority of elderly volunteer participants in the present study were also enrolled in these two already-published studies, we cannot affirm that the prominent results obtained in these previous studies had a direct impact on the data obtained in the present study. For instance, it is broadly accepted that the systemic antibody response does not show a direct impact on the mucosal antibody responses [54,55,56]; thus, we can putatively suggest that the increased specific-IgA levels for the influenza virus vaccine found by Monteiro and cols. [14] did not impact on the elevation of both total and specific-SIgA for the influenza virus vaccine observed in the present study.

Regarding the Gln effects on the immune response, it is well known that this non-essential amino acid can act not only as metabolic fuel and a precursor to protein synthesis, but also as a modulator of gene expression and activation of signaling pathways, especially in terms of cytokines [16]. Based on its prominent action in the modulation of the cytokine production on the mucosa, the overwhelming majority of data are related to the gut mucosa, and it was reported that Gln has an anti-inflammatory effect through an increase in local levels of IL-10, a classical anti-inflammatory cytokine [57]. In relation to the mucosa of the upper airway, it has been demonstrated that the oral Gln supplementation showed the capacity to modulate the production of pro- and anti-inflammatory cytokines by elevating the salivary levels of IL-10 and decreasing the TNF-alpha after an exhaustive exercise session [58]. However, Almeida et al. [15] recently showed that Gln supplementation for 30 consecutive days did not change the salivary levels of IL-10 and TNF-alpha in an elderly population.

Interestingly, our findings showed that elderly physically active subjects supplemented with Gln presented higher salivary levels of IL-10 after the supplementation period than the baseline levels. In addition, it was observed that the Gln supplementation increased the salivary levels of IL-6 and IL-17 in the same group of elderly subjects, in which IL-10 levels were higher.

There is a handful of evidence showing that the mucosal production of SIgA is regulated by a local Th2 immune response, since it was demonstrated that the presence of Th2 cytokines, such as IL-4, IL-5, IL-6, IL-10, and IL-13, can promote the differentiation of immature B cells into IgA- secreting plasma cells, which are responsible to secret SIgA in the mucosa [59]. Although we did not evaluate the salivary levels of IL-4, IL-5, and IL-13, the measurement of IL-6 and IL-10 is representative of the Th2 immune response profile and can be involved in the SIgA production. In this sense, here, we observed that the positive correlation between these cytokines was present in all time points studied regardless of supplementation, suggesting that this immune profile is important to elicit the mucosal protection by SIgA [59].

Especially in this study, the elderly group supplemented with Gln showed a significant increase of IL-6 and IL-10 as compared to the baseline values. This finding corroborates former studies, in which the Gln supplementation increases the Th2 response, by elevation of the levels of IL-6 and IL-10, in the mucosa of mice infected with bacteria or viruses [60,61]. It is of utmost importance to highlight that before the supplementation period, the volunteer groups presented similar levels of IL-6 and IL-10; thus, these observations can reinforce the studies mentioned above [60,61], since they demonstrated that only oral Gln supplementation was able to increase these cytokines in upper airway mucosa. Based on these pieces of information, we can suggest that the Gln supplementation improved the activation of a Th2 immune response, which consequently leads to the enhancement of the IgA-secreting plasma cells and, consequently, the SIgA levels in the elderly group supplemented with this amino acid.

In spite of there being no doubt that IL-6 and IL-10 can drive the Th cells towards a Th2 profile, it is widely accepted that IL-6 is closely involved in the up-regulation of both anti- and pro-inflammatory cytokines, such as IL-10 and IL-17, respectively [62]. At this point, it is worth clarifying that IL-6 and TGF-beta are necessary to promote the differentiation of Th cells to the Th17 immune profile, which, in a general way, is related to the immune response against extracellular and intracellular pathogens, and also in the inflammatory response [62,63]. However, concerning the mucosal immune response, the increase of IL-17 levels, the main cytokine involved in the Th17 profile, can improve the mucosal protection by eliciting the production and release of SIgA since mice deficient in IL-17 receptors showed a reduction of SIgA [59]. In this respect, it was reported that the binding of IL-17 to its receptor activates the NF-κB pathway, which leads to an increase in polymeric immunoglobulin receptor (pIgR) expression, an essential molecule involved in the transport of SIgA in the mucosa [59].

The remarkable Th17 profile in the mucosal protective immunity can be emphasized in terms of influenza infections due to its neutralization decreased the immune response against nasal influenza virus vaccination [63,64,65]. Corroborating these data, the increase of salivary IL-17 levels in the elderly supplemented with Gln post-vaccination can be putatively involved in the enhancement of mucosal protection associated with the SIgA levels, especially by the higher specific-SIgA levels for influenza virus vaccination in this elderly group. According to the literature, it was demonstrated that Gln metabolism has an impact on the Th cell differentiation, leading to a Th17 profile [66,67,68], and as previously cited, the IL-17 cytokine acts increasing the pIgR expression, which favors the transport of SIgA in the mucosa, consequently elevating its levels in this site. Taken into account the former data, the significant elevation of IL-17, a corollary cytokine of the immune response in the mucosa against influenza virus vaccination, elicited by Gln supplementation, could be associated with direct action of this amino acid on Th cells differentiation towards the Th17 profile or indirectly by the increase in salivary IL-6 levels. This last suggestion is based on our observation that the group supplemented with Gln presented positive correlations between IL-17 and IL-6, as well as between IL-17 and IL-10.

Another point to be highlighted is related to the observation that salivary IL-37 levels were significant reduced after influenza virus vaccination in the elderly groups regardless of the supplementation. This novelty shows that the physically active elderly preserves the capacity to modulate its mucosal immunity to respond against the influenza virus vaccination, since IL-37 is a well-known immunosuppressive cytokine. In agreement with the literature, IL-37, also named IL-1F7, is a member of the IL-1 superfamily, which differs from other IL-1 cytokine members due to the prominent property to suppress the immune responses (both innate and adaptative) and also to decrease inflammation [69,70,71,72]. In this way, studies have demonstrated that this cytokine is very important in the mucosal inflammatory responses, for instance, by controlling the exacerbated inflammation found in asthma and allergic rhinitis [73,74]. Corroborating this action to control the inflammation, we observed that IL-37 had a negative correlation with IL-10 in both elderly groups regardless of the time point evaluated, but it is noteworthy to mention that in the elderly group supplemented with placebo, the negative correlation showed a significant value. Therefore, the presence of salivary IL-37 in the elderly subjects shows that there was a modulation of immune/inflammatory responses in the oral cavity before the vaccination and, interestingly, its reduction post-vaccination can help in the induction of a protective immune response in this site against the influenza virus vaccination.

## 5. Conclusions

Taken together, the results of this study have shown, for the first time, that physically active elderly subjects supplemented with Gln presented an elevation of total and specific-SIgA levels against the influenza virus vaccination in response to the modulation of Th cell profile towards not only to the Th2 profile but also the Th17 immune response profile.

## Figures and Tables

**Table 1:** Physical and anthropometric characteristics of the elderly subject groups supplemented with placebo or L-glutamine.

Characteristics	Volunteers (*n* = 83)		*p* Value
	Placebo (*n* = 41)	L-Glutamine (*n* = 42)	
Age (year)	73.3 ± 6.4	71.8 ± 5.7	>0.05
Women (*n*)	33	32	>0.05
Men (*n*)	8	10	>0.05
Sex ratio (M:W)	1:4.125	1:3.2	>0.05
Height (m)	1.56 ± 0.09	1.58 ± 0.09	>0.05
Weight (kg)	63.5 ± 10.5	67.3 ± 14.6	>0.05
Body mass index (kg/m^2^)	26.3 ± 3.8	26.5 ± 4.1	>0.05
Total body fat (%)	37.3 ± 8.9	36.3 ± 8.2	>0.05
Fat-free mass (%)	63.1 ± 8.5	63.9 ± 8.1	>0.05
Skeletal muscle mass (kg)	19.1 ± 3.8	20.6 ± 4.1	>0.05
IPAQ			
Physical activity (min/week)	664 ± 144	696 ± 182	>0.05
Sitting (min/week)	1453 ± 579	1557 ± 527	>0.05

Note: IPAQ-International Physical Activity Questionnaire.

## Data Availability

The data presented in this study are available on request from the corresponding author. The data are not publicly available due to privacy and ethical restrictions.

## Supplementary Materials

The following are available online at https://www.mdpi.com/2076-393X/9/2/107/s1, Table S1: Pearson’s correlation analysis of the salivary levels of pro- and anti-inflammatory cytokines in the elderly subjects groups supplemented with placebo or L-glutamine pre (before) and post 30 days of Influenza virus vaccination and supplementation.
