# Supplementary material for: Elderly Subjects Supplemented with L-Glutamine Shows an Improvement of Mucosal Immunity in the Upper Airways in Response to Influenza Virus Vaccination
Source: Vaccines (Basel). 2021 Jan 31;9(2):107. doi: 10.3390/vaccines9020107 (PMC7911866; doi:10.3390/vaccines9020107)
Supplement: Supplementary file 1 [file vaccines-09-00107-s001.pdf]

Table S1: Pearson's correlation analysis of the salivary levels of pro- and anti-inflammatory cytokines in the elderly subjects groups supplemented with placebo or L-glutamine pre (before) and post 30 days of Influenza virus vaccination and supplementation.

Groups<br>Variables	Placebo (n=41)<br>Pre	 Post	L-glutamine (n=42)<br>Pre	 Post
IL-6/IL-10	*r*= **0.958**<br>***p<0.0001***	*r*= **0.985**<br>***p<0.0001***	*r*= **0.999**<br>***p<0.0001***	*r*= ***0.995***<br>***p<0.0001***
IL-6/IL-17	*r*= -0.314<br>*p=0.154*	*r*= -0.071<br>*p=0.763*	*r*= 0.333<br>*p=0.117*	*r*= **0.889**<br>***p<0.0001***
IL-6/IL-37	*r*= -0.312<br>*p=0.157*	*r*= -0.263<br>*p=0.236*	*r*= -0.305<br>*p=0.218*	*r*= -0.298<br>*p=0.230*
IL-6/TNF-α	*r*= -0.163<br>*p=0.329*	*r*= 0.085<br>*p=0.612*	*r*= -0.063<br>*p=0.694*	*r*= -0.060<br>*p=0.709*
IL-10/IL-17	*r*= -0.315<br>*p=-.154*	*r*= -0.199<br>*p=0.638*	*r*= 0.326<br>*p=0.187*	*r*= **0.874**<br>***p<0.0001***
IL-10/IL-37	*r*= **-0.445**<br>***p=0.038***	*r*= **-0.459**<br>***p=0.031***	*r*= -0.293<br>*p=0.239*	*r*= -0.300<br>*p=0.226*
IL-10/TNF-α	*r*= -0.183<br>*p=-0.272*	*r*= -0.086<br>*p=0.629*	*r*= -0.060<br>*p=0.712*	*r*= -0.058<br>*p=0.718*
IL-17/IL-37	*r*= 0.210<br>*p=0.348*	*r*= 0.014<br>*p=0.952*	*r*= -0.193<br>*p=0.443*	*r*= -0.057<br>*p=0;822*
IL-17/TNF- α	*r*= -0.070<br>*p=0.758*	*r*= 0.191<br>*p=0.887*	*r*= -0.014<br>*p=0.641*	*r*= 0.165<br>*p=0.651*
IL-37/IL-17	*r*= 0.210<br>*p=0.348*	*r*= 0.014<br>*p=0.952*	*r*= -0.193<br>*p=0.443*	*r*= -0.057<br>*p=0.822*
IL-37/TNF-α	*r*= 0.260<br>*p=0.242*	*r*= 0.191<br>*p=0.394*	*r*= -0.014<br>*p=0.958*	*r*= 0.165<br>*p=0.527*
